# Effective approaches to public involvement in care home research: a systematic review and narrative synthesis

**DOI:** 10.1186/s40900-023-00453-2

**Published:** 2023-06-02

**Authors:** Tanisha Burgher, Victoria Shepherd, Claire Nollett

**Affiliations:** 1 School of Medicine, Cardiff University, Cardiff, UK; 2 Centre for Trials Research, Cardiff University, 4Th Floor, Neuadd Meirionnydd, Heath Park, Cardiff, CF14 4YS UK

**Keywords:** Care home residents, Disabled adults, Long-term care facilities, Nursing homes, Older adults, Person-centred opportunities, Public involvement, Stakeholder involvement, Systematic review

## Abstract

**Background:**

Public involvement (often referred to as patient and public involvement or PPI) integrates the voices of the public in health and care research. However, groups such as care home residents are often excluded from involvement opportunities due to the complexities of involving people with additional care and communication needs. Despite a range of approaches being used, there is little understanding about how best to incorporate their experiences, and those of other care home stakeholders, into the design and conduct of research.

**Objective:**

A systematic review was conducted to identify PPI methods that better meet the specific needs of care home stakeholders. This was undertaken by (1) outlining effective PPI approaches used in care home research and the key stakeholders involved; (2) describing the role of PPI in different care home contexts and (3) identifying stakeholders’ experiences and attitudes towards PPI in care homes.

**Methods:**

Databases CINAHL, Embase, MEDLINE, PsycINFO and Scopus were searched for English language papers from inception to November 2021. A narrative synthesis approach was utilised to organise the extracted data into five themes.

**Results:**

The search initially yielded 2314 articles (following de-duplication), with 27 meeting the inclusion criteria. Articles reported a range of input from stakeholders (including residents, staff, relatives and community stakeholders), with the impact of PPI varying according to the type of care establishment and research context. The experiences and reflections of stakeholders’ about their involvement in care home research varied, with some studies offering first-hand accounts compared with summaries from researchers. Some articles explicitly evaluated the effectiveness of the PPI approach using specific outcome measures whilst others indirectly described the impact of their approach. Five themes were identified as characterising an effective PPI approach: (1) valuing stakeholders’ perspectives, (2) awareness of the multi-faceted research context, (3) ensuring inclusivity and transparency, (4) maintaining flexibility and adaptability and (5) utilising resources and wider support.

**Conclusion:**

Effective PPI in care home research requires researchers to create person-centred opportunities to adequately involve groups with physical and cognitive impairments. The findings led to the creation of evidence-based practical recommendations to support future involvement opportunities and help researchers develop strategies for inclusive opportunities for involvement.

*Systematic Review Registration*: The review was prospectively registered on PROPSERO (CRD42021293353).

## Abstract (plain language summary)

Public involvement actively incorporates the views and lived experiences of those affected by research and is essential to ensure that research is meaningful. However, involving some under-served groups, such as people living in care homes, can be complicated due to their additional care and communication needs. The best way to involve care home residents, families and staff in research is unclear. In this systematic review we looked at studies using different approaches to public involvement in care home research. We found that studies involved different combinations of residents, families, and staff depending on the type of study and what was involved. Some articles described what methods of public involvement had taken place, whilst others explained how effective their approach to involvement had been and why. For involvement to be effective, it was important that researchers valued differing perspectives by providing a safe forum for stakeholders to share their opinions. Researchers should also understand that successful involvement requires flexibility and adaptability to accommodate the varied needs of individuals such as time commitments and simplifying the complexities of the research terminology. Additionally, researchers should utilise the wide range of available resources and support to ensure under-served groups are appropriately included within the research team. We concluded that public involvement in care home research needs to focus on the needs of the individuals to ensure that people with physical and cognitive impairments can be involved. The findings can help care home researchers to create equal opportunities for all to be involved.

## Background

Public involvement (often referred to as patient and public involvement or PPI) has evolved into an essential aspect of research practice, which endeavours to integrate the voices of the public into the research process [1–3]. The National Institute for Health Research (NIHR) defines PPI in research as, “*research carried out ‘with’ or ‘by’ members of the public rather than ‘to’, ‘about’ or ‘for’ ‘them*” [1]. The public are ‘experts by experience’, with vast experiential knowledge that offers a specific perspective to research [1, 4]. The NIHR is the largest funder of health and care research in the UK; specifically advocating for PPI in research with particular interest in the involvement of marginalised groups [3].

Despite this, care home residents continue to be under-represented, with minimal understanding of the benefits of utilising their first-hand experiences in research [2, 3]. Older adults account for the largest growing segment of the population, of which approximately half a million live in 19,000 care homes in the UK [1, 5]. Younger adults aged 18–64 who have learning disabilities, mental health problems and other social needs may also live in residential care. Younger adults represent one-third of care users in the UK which accounts for over half of local authority spending [6]. However, historically there has been much less research conducted within these communities compared to individuals within hospital settings; with further disparities between the care received from older people in social care compared to younger adults [1, 6, 7]. ‘Care home’ is commonly used as a catch-all term which incorporates nursing and residential homes and is defined by Luff et al. [8] as “all residential long-term care settings which provide group living and personal and/or nursing care for older people and other adults”.

Adults dependent on social care are the greatest recipients of healthcare services, with many experiencing complex multimorbidity, increasing frailty and dependency on nursing staff [5, 9]. Care home residents are an under-served group in research partly due to communication challenges with hearing, visual and cognitive impairments which presents methodological challenges including difficulty obtaining informed consent, the additional time needed to support participation, challenges in securing funding, and lack of expertise in research involving these groups [4, 10]. The absence of representative and inclusive research in this population group can result in findings that are biased and mismatched to the needs of care home residents; thus, precipitating ineffective treatments, divergent agendas and misrepresentation [11].

When designing and conducting research, it is important embed public involvement throughout which incorporates the lived experience of care home residents and caregivers [4, 12] alongside the scientific knowledge of academic researchers. This involvement can be viewed as a continuum ranging from individuals being consulted about their views and opinions through to being co-researchers, co-producers, or as project leaders of the research [13, 14]. They can become active partners within the research design, delivery, data analysis and dissemination [1, 13, 14]. Whilst there is there is a growing recognition of the value of PPI in care home research, at present, there is insufficient knowledge about effective strategies to involve vulnerable adults as research partners with meaningful impact [15], particularly in care home settings. Previous reviews have focused solely on the involvement of care home residents [16], however care homes can be viewed as ‘communities of care’ and so the perspectives of other stakeholders are often involved. An understanding of how best to involve multiple stakeholders, who will have a range of roles and needs, and in different types of research and care home contexts has yet to be explored.

To address this gap, we aimed to systematically identify and synthesise published studies to identify effective PPI approaches used in care home research. The objectives were to: (1) outline what approaches were used in PPI in care home research and the key stakeholders involved; (2) describe the role of PPI in different care home contexts and (3) identify stakeholders’ experiences and attitudes towards PPI in care home research. In this review, ‘public’ refers to residents (older people and adults with disabilities dependent on social care), relatives, caregivers and representative organisations. These groups can be considered key stakeholders in care home research. Approaches were considered ‘effective’ where the researchers who reported the study had viewed their experience of PPI activity as positive or having been successful in achieving its aims.

The findings from this systematic review can be used to improve the standard of PPI in care home research by identifying the best approaches to create inclusive opportunities for care home stakeholders [5]. Better understanding about how to involve key stakeholders in care home research will enhance the quality of studies being conducted, ensure that health and care research is meaningful and leads to improvement in the care that these groups receive, and help to address the challenges of social exclusion, injustice, and marginalisation that members of these groups can experience [4, 5].

## Methods

This systematic review is reported in accordance with the 2020 Preferred Reporting Items for Systematic Reviews and Meta-Analyses (PRISMA) guidelines [17]. The protocol was prospectively registered in the PROSPERO 2021 database (CRD42021293353). A narrative synthesis was adopted as it was likely there would be wide heterogeneity between the studies. This approach uses data to ‘tell a story’ and was guided by the Cochrane Collaboration and Economics and Social Research Council [18, 19].

### Eligibility criteria

This review was limited to English language studies without date limits on the publication year. Studies were included if they reported PPI research in care homes, residential homes, or nursing homes regardless of the care home population (older people or younger adults with disabilities), research topic or study methodology. Studies were excluded if they did not report PPI in care home research, key stakeholders were not included, or the study was conducted in other social care settings. The SPIDER framework (Setting, Phenomenon of interest, Design, Evaluation, Research design) [20] was utilised to develop the eligibility criteria (Table 1)*.* This approach is best suited for qualitative research and enables the exploration of behaviours and individual experiences.

**Table 1:** Study eligibility criteria

SPIDER framework	Inclusion criteria	Exclusion criteria
Setting	Care homes Residential homes Nursing homes	Social care services which include Other supported accommodation Other forms of social support
Phenomenon of Interest	Stakeholders’ experiences and attitudes to PPI in care home research Stakeholders include: Care home residents and potential care home residents Care home staff and managers Informal (unpaid) carers Parents/guardians Organisations that represent people who use care homes	Studies which focus solely on the attitudes of people in settings outside the care home
Design	Reports, evaluations and reflections	Secondary data including systematic reviews and literature reviews
Evaluation	Exploration of approaches to PPI in care home research	
Research Design	Qualitative and mixed methods: Randomised controlled trials Case–control studies Observational studies Qualitative studies Cohort studies Non-randomised studies	Studies reporting on PPI methods not related to care-home research Absence of empirical research data (editorial and protocol) Systematic reviews and literature reviews

*PPI* patient and public involvement

### Systematic search strategy

A search of five electronic databases (CINAHL, Embase, MEDLINE, PsycINFO and Scopus) was conducted in November 2021. The search strategy was developed with guidance from a subject librarian (Appendix 1) to capture the three key concepts of the research question comprising (1) PPI in research, (2) care homes and (3) attitudes and approaches. The search strings were adapted from NIHR recommendations on search terms for ‘public involvement’ [21], and two systematic reviews exploring end-of-life care in care homes [22] and attitudes and approaches to PPI in research [23]. Boolean search terms ‘OR’ and ‘AND’ were used to translate the research question into research string that captured the relevant articles from bibliographic databases.

A supplementary lateral search of additional literature resources was conducted by searching reference lists of studies and applying comprehensive pearl-growing techniques to broaden the search through forward citation of included studies (completed February 2022). Additional studies were retrieved from web searching and searching a topic-specific journal (Research Involvement and Engagement) by adapting the search concepts used in the electronic databases to create specific search strategies for additional literature resources.

### Study selection

De-duplicated studies were exported into EndNote 20.2. Study selection comprised of three stages: firstly, the titles and abstracts from the initial literature search were screened by the first author. Of these studies, 10% were then double screened by another researcher to ensure consistency. Secondly, the full text of included articles (n = 94) were independently reviewed by two co-authors for eligibility, the reasons for exclusion were recorded in accordance with the PRISMA guidance [17]. Records where the full text was not retrievable were considered ineligible. Thirdly, disagreements over study eligibility of those classified as ‘maybe’ (n = 28) and ‘conflict’ (n = 15) were resolved by reviewing the full text of the disputed articles and comparing the decision-making of included articles with the disputed articles through transparent discussion. A third researcher was consulted where necessary to reach a consensus and develop a clearer criterion to reduce uncertainty going forward.

### Critical appraisal

In accordance with the published protocol, quality assessment of included studies was attempted using the Mixed Methods Appraisal Tool (MMAT) 2018 version which enables appraisal of different study designs [24]. However, during the critical appraisal process, the MMAT was found to be inappropriate due to the lack of consistency of PPI reporting and absence of established methodological rigour. A review of other appraisal tools failed to identify an alternative appropriate tool for assessing the reporting of PPI. Hence, studies were not excluded based on methodological quality but the issues that arose with the reporting of PPI in research were explored in the data synthesis stage as recommended in the narrative synthesis guidance.

### Data extraction

A data extraction tool was developed for this review with guidance from Backhouse et al. [3]. Extracted data included study details, research methodology, recruitment, the barriers and elements enhancing PPI in research (Appendix 2). All data extraction was conducted by the first author, with double data extraction of 10% of studies performed by the other two authors. Due to the variability and heterogeneity of the PPI reporting within the included articles, the regular discussions with the research team during the study selection led to a robust framework when standardising subjective assessment. The data extraction forms of the included articles were imported into NVivo 12 software to aid thematic code generation.

### Data synthesis

Codes were created to capture the meaning of key underlying and recurring concepts and were organised into themes which were iteratively developed using headings from the data extraction tool. Definitions for each generated theme were developed and refined through regular discussions amongst the research team.

## Results

### Systematic search

**Fig. 1:**
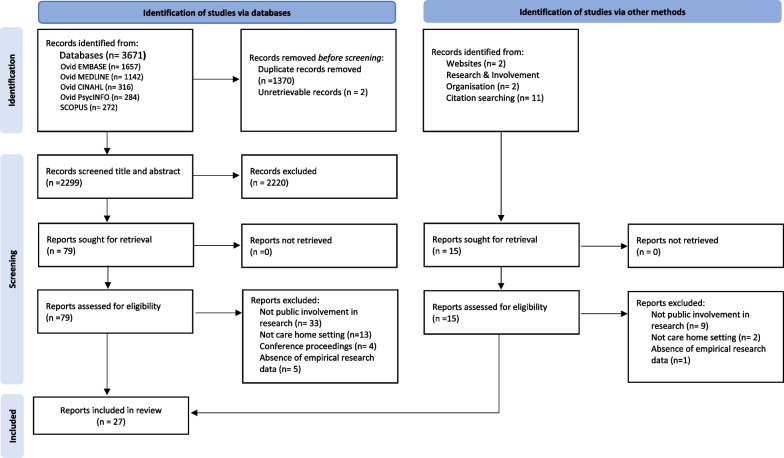
PRISMA flow diagram

Database searches yielded 3671 papers with an additional 15 papers identified from other sources. Following de-duplication this resulted in a total of 2314 records, of which 94 studies were retrieved for full-text assessment following identification of studies via databases (n = 79) and other methods (n = 15) (Fig. 1, PRISMA Diagram). Using the inclusion criteria, 27 studies were subsequently included in the analysis.

The characteristics of the studies are reported in Table 2. Most studies referred to older people but varied in study topic and degree of stakeholder involvement. Only one study [25] focused on younger adults with disabilities living in care homes. Study locations included the UK (n = 13), Europe (n = 5), Canada (n = 5), USA (n = 3) and South America (n = 1).

**Table 2:** Study summary characteristics

Study author	Country	Study type and topic	Type of PPI^a^ (PPI terminology used by authors)	Characteristics of care homes (n = care homes involved)	PPI members and stakeholders	Characteristics of PPI members and stakeholders (n = PPI stakeholders involved)
Aubrecht et al. [25]	Canada USA	Commentary on the lived experiences of disabled adults living in long-term care homes	Partnership (co-researchers, co-authors)	Residential long-term care (n = 3)	Disabled residents and policy decision-makers	Female residents between 28 and 48 years with physical disabilities (cerebral palsy, multiple sclerosis, muscular dystrophy) (n = 3)
Brown et al. [26]	UK	Critical reflections to develop recommendations to optimise effective PPI in research	Partnership (community representatives)	Care home (n = not specified)	Older members of the public	Community representatives of White British ethnicity (1 female was 74 years and 2 males were 56 and 84 years) (n = 3)
Burns et al. [27]	UK	Case study^b^ exploring how participatory organisational research amplifies the voices of older people	Partnership (co-produced)	Care home (n = 8)	Residents, relatives, friends, care assistants, nurses, care managers, care homeowners and community stakeholders	5 residents, all aged 85 and over and 4 relatives of residents living in care homes) (n = 9)
Chamberlain et al. [28]	Canada	Priority setting methods to engage citizens and stakeholders to improve the quality of care and life in long-term care settings	Consultation (team members, citizen engagement)	Long-term care home (nursing home, personal care homes) (n = 97)	Voices Of Individuals, family and friend Caregivers Educating uS (*VOICES*) Residents, future residents, family/friend caregivers, decision-makers and long-term care owner-operators	9 VOICES members, 1 direct care provider, 1 provincial ministry of health representative, 2 long-term care owner-operators, 4 provincial health authority representatives, and 2 provincial association representatives (n = 19)
Charles et al. [29]	Canada	Case study^b^ of an intervention development to measure resident care requirements in the nursing home	Placation (research transfer agents)	Long-term care home (nursing home) (n = 1)	Long-term care facility owners, operators, care providers and facility staff	Not specified
Eisenstein et al. [30]	USA	Process evaluation focused on older people and their caregivers’ experiences within research	Partnership (Sages)	Skilled nursing facility (a short-term, long-term care and dementia care facility) (n = 1)	Bureau of Sages Community representatives, researchers, healthcare professionals	4 Lieberman community members, 5 stay-at-home members, 6 researchers/healthcare professionals (n = 15)
Elliott et al. [31]	Canada	Commentary on the development of COVID-19 research aims with older adults	Partnership	Community-dwelling Assisted living accommodation (n = not specified)	Seniors helping as research partners (*SHARP*) Community-dwelling older adults and family caregivers who provide support to older adults	SHARP members, the majority of White British ethnicity, all aged over 65 years (12 females and 6 males) (n = 18)
Evans et al. [32]	UK	Evaluation on the role of older people in research to improve the standards of care and quality of life for care home residents	Partnership (community researchers and evaluators)	Care homes (n = 10)	Partnerships for older people project (*POPPs*) Older people, Representatives from Gloucestershire Primary Care Trust, Gloucestershire County Council and Gloucester Older Persons’ Assembly	Community representatives (n = 4)
Froggatt et al. [33]	UK	Evaluation of the integration between primary health and care home service provision	Partnership	Care homes (n = 6)	Individuals with prior experience engaging with care home staff and residents	Not specified
Goodman et al. [34]	UK	Longitudinal mixed-methods study exploring the experiences of living and dying of older people living in care homes	Partnership (co-researchers, critical friend)	Care homes (n = 3)	Public Involvement in Research Group (PIRg) Family/friend caregivers	PIRg members (n = 4)
Griffiths et al. [35]	UK	Clinical service evaluation focused on maintaining and improving mouth care for care home residents	Partnership (cooperation, co-production)	Care homes (n = 2)	Care home nurses, care workers, managers and specialist colleagues with experience in dentistry, behaviour and systematic reviews	Care home staff (n = 8)
Hewitt et al. [36]	Guyana	Process evaluation of an intervention development to improve the diet, health and quality of life in a residential home	Consultation (focus group discussions and informal conversations)	Non-profit residential home (n = 1)	Residents, domestic staff, residential home management and community stakeholders	Residents of African descent, between 73 and 99 years. (10 females and 4 males) (n = 14)
Hoffman et al. [37]	USA	Intervention development to facilitate shared decision-making for older adults and their families	Partnership (co-production, codesign, stakeholder advisory panel)	Aging resource centre (n = 1)	Stakeholder advisory panel Older adults, caregivers, occupational specialists	Members of the stakeholder advisory panel (2 older adults, 2 family caregivers, 2 decision scientists, 4 informaticians, 3 geriatric psychiatrists and 3 memory care specialists) (n = 16) An additional 12 older adults and caregivers involved at later stages in the project
Johannessen et al. [38]	Norway	Mixed-methods study of an intervention development to improve quality and safety in nursing homes	Placation (co-researchers, future users)	Nursing homes (n = 2)	Nurse counsellors, PPI representatives, patient ombudsman and managers of nursing homes and home care	Co-researchers (n = 7)
Killett et al. [39]	UK	Organisational factors associated with mistreatment of older people in care homes	Consultation and Placation (peer researchers, key informants)	Residential care home (n = 8)	Residents, family caregivers and older people with personal experience of care homes	Not specified
Logan et al. [40]	UK	Multicentre, cluster randomised controlled trial of an intervention development to prevent falls in older people in care homes	Partnership (co-design, hub-and-spoke approach)	Care homes (n = 10)	*Hub PPI member*: Former caregiver for a care home resident *Spoke PPI members:* care home manager, family caregiver, patient representative and associated stakeholders	1 Hub PPI member 4 Spoke PPI members: retired care home manager (female), retired medic (male), carer for a relative with dementia (male), patient research ambassador and a lay chairperson for NIHR (female) (n = 5)
Mann et al. [41] (Findings and direct quotes from Chamberlain et al. 2020)	Canada	Reflections on utilising priority setting methods to engage citizens and stakeholders to improve the quality of care and life in long-term care settings	Consultation (team members, citizen engagement)	Long-term care home (nursing home, personal care homes) (n = 97)	Voices Of Individuals, family and friend Caregivers Educating uS (*VOICES*) Residents, future residents, family/friend caregivers, decision-makers and long-term care owner-operators	VOICES members: 1 direct care provider, 1 provincial ministry of health representative, 2 long-term care owner-operators, 4 provincial health authority representatives, and 2 provincial association representatives (n = 9)
Oude et al. [42]	Netherlands	Context mapping study exploring the impact of losing items and assistive devices in nursing homes	Placation (expert, co-design)	Nursing homes (n = 2)	Nursing home workforce	Participants’ average number of years active in the workforce 21 years (12 females, mean age = 47 years) (n = 13)
Scheffelaar et al. [43]	Netherlands	Case study^b^ evaluating the quality-of-care relationships between service users and care professionals in long-term care	Partnership (co-researcher, co-author)	Long-term care facilities (n = 3; older adults, mental health, and intellectual disability teams)	*Co-researchers*: residents *Stakeholders:* representatives of care providers and branch organisations, nationwide client council organisations, staff from care organisations and health insurers	Co-researchers who were physically frail (3 females and 2 males) (n = 5)
Shura et al. [44]	USA	Participatory action research to promote change in the culture of long-term care facilities	Placation (co-researcher, experts)	Continuing care retirement community (n = 4 units; assisted living, nursing home and 2 specialised memory support)	Research groups: residents, family caregivers and care home staff	7 research groups (2 assisted living units and 5 nursing home units). Each research group had 4–7 residents, 1–2 family members and 1–3 staff (n = 75)
Smith et al. [45]	UK	Retrospective reflections on engaging care home managers and older adults in research	Partnership (research/study advisory group)	Care homes (Study 1 = 6 homes and Study 2 = 34 homes)	Research advisory group: care home managers, representatives from local authorities, PPI representatives, and the Care Quality Commission	Care home managers (n = 30)
Stocker et al. [46]	UK	Reflections on the impact of PPI in care home research	Consultation (PPI partners)	Care home (n = 1)	University-supported Care Home Interest Group (CHIG) Health-care professionals, care home staff, local authority staff, clinical governance/research support roles	Workshop 1: 7 CHIG members Workshop 2: 4 CHIG members (n = 8)
Stockigt et al. [47]	Germany	Evaluating the stakeholder experience of an intervention development for the use of soft physical touch to enhance wellbeing of elderly patients	Consultation (stakeholder involvement)	Nursing home (n = 1)	Patients with chronic pain, nurses, experts of therapies using physical touch, and staff of the Institute of Social Medicine of the Charité and the Centre for Quality in Care	Stakeholders (n = 18)
Walsh et al. [48]	UK	Mixed-methods study of an intervention development targeting antipsychotic prescribing for nursing home residents with dementia	Consultation (advisory groups)	Nursing home (multisite study)	Residents living with dementia, health and social care professionals, family caregivers, advocacy groups and academics	Advisory group members = 4 residents (2 females, 2 males) and 2 female family caregivers (n = 6)
Walshe et al. [49]	UK	Four-stage process of an Intervention development to utilise Namaste Care in the nursing home setting	Partnership (PPI representatives, co-design)	Nursing home (n = not specified)	Nursing care home staff (includes managers, nurses, care assistants, activity coordinators or volunteers), family members/carers and PPI representatives	18 staff, 1 volunteer and 5 PPI members (n = 24)
Willis et al. [50]	UK	Reflections of the co-production of research promoting LGBT inclusion in care homes for older people	Partnership (co-production, community advisors)	Residential Care homes (n = 6)	*Community advisors:* LGBT volunteers and ally from the local community	Community advisors aged 35–65 years of variable ethnic backgrounds, White British, Jewish, British-Asian and Bangladeshi. There were 2 Lesbian, 3 Gay, 1 Queer and 1 Transgender volunteer (n = 8)
Woelders et al. [51]	Netherlands	Participatory action research care home engaging elderly care residents in research via dialogue approaches	Partnership	Residential Care home (n = 1)	Residents, professional team leaders and spiritual counsellors (facilitators)	2 males facilitators aged 25 and 50 years 10 residents aged between 67 and 95 years (2 males and 8 females) with physical impairments including immobility, hearing and visual problems and loss of memory (n = 12)

*CHIG* Care Home Interest Group, *LGBT* lesbian, gay, bisexual, and transgender, *NIHR* National Institute for Health Research, *PIRg* Public Involvement in Research Group, *POPPs* partnerships for older people project, *PPI* patient and public involvement, *SHARP* seniors helping as research partners, *UK* United Kingdom, *USA* The United States of America, *VOICES* voices of individuals, family and friend caregivers educating us

^a^The type of PPI stakeholder involvement was categorised using the typology outlined by Sherry Arnstein in the Ladder of Citizen Participation [52]

^b^Case study: an in-depth exploration of issues central to a particular decision-making process [24]

### Synthesis of findings

An effective approach to PPI was defined by reviewing stakeholders’ experiences and attitudes to PPI in care home research as having been positive or achieving its aims. Effective approaches to PPI in care home research were grouped into five themes: valuing stakeholders’ perspectives; ensuring inclusivity and transparency; awareness of the multi-faceted research context; maintaining flexibility and adaptability and utilising resources and wider support. Table 3 illustrates the overarching themes with associated definitions and examples from the included studies.

**Table 3:** Definitions of overarching themes and associated examples

Overarching themes	Example
*Valuing stakeholders’ perspectives* There must be a clear recognition that the lived experiences and personal viewpoints of PPI stakeholders are pivotal to the research design and outcomes, including the dissemination and impact to clinical practice	“The PPI team were instrumental in helping the research team to secure additional funding, in helping with our funding monitoring committee and in adapting our recruitment methods. The PPI team suggested that care home managers were best placed to understand their residents’ wishes in terms of participating in this research, which resulted in a 3% increase in recruitment per care home.” [40]
*Inclusivity and transparency* Creating a research environment of inclusivity and transparency enables diverse stakeholder groups to be involved in research; via a spectrum of involvement which provides a safe communicative space for PPI stakeholders to build a rapport with the research team	“Senior management was willing for staff to be involved in all aspects of the research, including meetings, completing questionnaires and working alongside researchers to develop the grant application.” [35]
*Multi-faceted research context* An effective PPI approach considers the complexity of the research topic in relation to the existing knowledge of PPI stakeholders, balances the power dynamic hierarchies and encourages a representative recruitment approach	“We conclude that striving for the collective involvement of clients in residential care organisations is a complex and delicate process. It is not taking place in a vacuum, but is embedded in a socio-cultural, political context, related to power asymmetries.” [51]
*Flexibility and adaptability* An iterative and flexible approach to the methods of involvement, accommodates the diverse needs of PPI stakeholders and adapts to the preferred mode of communication and optimises accessibility	“It was important to consider the physical, social, and temporal environment to allow people with a range of physical and sensory constraints to communicate effectively and to enable discourse and meaning to develop. Older people living in residential care met with the members of the research team but without care staff present.” [27]
*Resources and wider support* The ability to tailor training to PPI stakeholders’ needs and offer additional financial aid, enhances the scale of collaboration, and impacts the research design	“A financial budget was available for paying co-researchers an allowance for their participation, but such an allowance was tied to national restrictions. These co-researchers are only allowed to receive 1500 euros per year for their volunteering work, otherwise the reimbursement will be deducted from their benefit resulting in extra bureaucracy.” [43]

*PPI* patient and public involvement

**Fig. 2:**
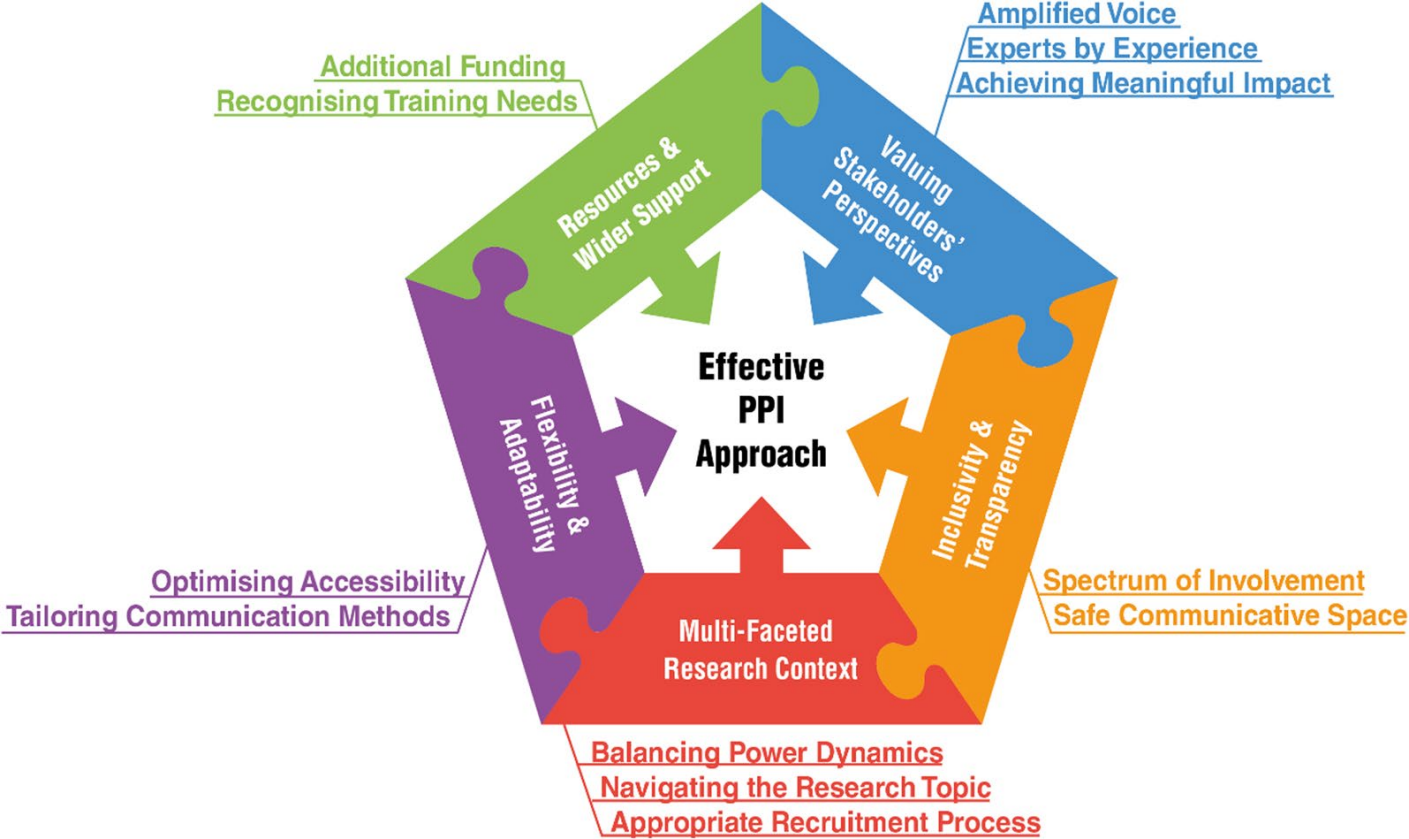
Conceptual map of an effective ppi approach in research

Each of the five overarching themes have associated subthemes which is illustrated in the conceptual diagram shown in Fig. 2*.*

### Valuing stakeholders’ perspectives

#### Experts by experience

For PPI in care home research to be effective, stakeholders should be valued as experts due to their authentic experiential knowledge. Most studies formulated inclusion criteria that encapsulated the first-hand experiences of stakeholders however physical impairments such as reduced sight, hearing and mobility challenged the involvement of residents [36, 38]. Despite this, one study defined residents as ‘visionaries’ and included residents regardless of physical or memory difficulties; empowering their role as active research partners [44]. Directly involving PPI stakeholders in the data analysis unsettled some researchers as they were reluctant to view PPI stakeholders as ‘experts by experience’ [36, 40, 50].

#### Achieving meaningful impact

The insights shared by PPI stakeholders should guide the research for meaningful impact [30]. Three studies centralised PPI stakeholders within the study design which strengthened the quality and relevance of the research methodology [25, 32, 34]. Another study utilised PPI stakeholders’ recommendations to involve care home managers in recruitment which ultimately led to a 3% increase in participant recruitment per home [40]. Despite this, the overall benefits of PPI approaches within research appeared to be superficial and ineffective in other studies [41, 44]. However, acknowledging the distinctive and valuable contribution of PPI stakeholders was regarded an effective PPI approach [32, 40, 49].

#### Amplified voice

Extending the influence of PPI stakeholders beyond the research design to dissemination promotes the resonance and impact of the research findings [45, 46]. As co-authors, PPI stakeholders provided a reflective perspective that was tailored to their specific needs by promoting conversations, facilitating group discussions and interviews, pilot testing of interventions with subsequent findings relayed to the academic team [37, 38, 50]. A user-centred approach empowers PPI stakeholders and offers quality assurance throughout the study, as the involvement is tailored to the specific needs and strengths of the PPI stakeholders. Furthermore, dissemination at national conferences offered an added sense of authenticity and credibility [27, 30, 37]. This amplified PPI stakeholder input improves pathways for future quality improvement projects by increasing the applicability of the research findings [35, 43].

### Inclusivity and transparency

#### Spectrum of involvement

PPI stakeholders had a variety of roles in studies. The continuum of involvement was vast including PPI stakeholder contribution at iterative stages of the research design [28, 32, 49] during intervention development [37, 47, 48], the production of study materials [25, 46], data analysis [29, 39, 43] and facilitating discussions [32, 50] which has been shown to enhance research outcomes. An effective PPI approach extends the role of PPI stakeholders throughout all aspects of research from encouraging attendance in face-to-face research meetings, asking for feedback on projects via preferred communication methods to contribution in NIHR-funded reviews [46]. This comprehensive involvement of PPI stakeholders enables distinct and often unheard perspectives to permeate research.

#### Safe communicative space

Inclusive and transparent communication between PPI stakeholders and researchers provided a safe space to share opinions and lived experiences [51]. This was achieved by hosting separate meetings for PPI stakeholders [48], eliminating academic jargon [44] to building a rapport and fostering professional relationships [33, 40] resulting in sustained willingness to participate [34, 35]. A safe communicative space enables PPI stakeholders to interact with academics with authenticity and ‘change the dynamic in a positive way’, nurturing enjoyable collaborations that extend beyond professional settings to informal social events [26].

### Multi-faceted research context

#### Balancing power dynamics

The hierarchical positioning between PPI stakeholders and researchers and between care home administration and residents was influential to PPI in care home research [26, 36]. Creating a power balance between researchers and PPI stakeholders enabled polyvocal perspectives, trust and openness [27, 28, 30, 43]. An imbalance was potentiated through language and discourse [51] and where the role of PPI stakeholders within decision-making was undefined [34, 41] or where tension was created between PPI stakeholders and researchers due to differing expectations about, and understanding of, research timescales [45, 48]. As a result, the views and inputs of residents were often overwhelmed and overpowered by multiple perspectives from clinical researchers, staff and even relatives. This highlights existing power dynamics and the complexities of addressing research translation and implementing research roles with PPI stakeholders [35]. An effective PPI approach addressed the inherent power relationships within research to encourage maximal involvement.

#### Navigating the research topic

Many studies noted that the greater the complexity and difficulty of the research concepts, the more disengaged and overwhelmed PPI stakeholders become. The vast amount of complex information led to projects sometimes being ‘out-of-scope’ for some stakeholders [28, 50] which was exacerbated through academic jargon and the pace of discussions [26, 48]. This cognitive burden was sometimes coupled with the physical demand of the research schedule [30, 36, 38] and emotional responses provoked by the research topic [33]. Effective PPI used lay summaries and language which connected with the initial knowledge of PPI stakeholders to create a comfortable research environment [40, 41].

#### Appropriate recruitment process

Utilising a variety of strategies to recruit PPI stakeholders and care organisations increased the generalisability of the findings [39, 42]. Advertisements, posters and flyers were distributed to PPI stakeholders via local publicity; using attractive taglines to engage specific stakeholder groups [32, 46, 51]. Existing recruitment structures were also exploited via volunteer forums, pre-established PPI groups and attendance to local stakeholder conferences [26, 40, 48]. Some studies adopted a top-down recruitment approach where PPI stakeholders were nominated and purposively selected [29, 38, 51]. Thus, appropriate consideration of recruitment methods increased the representativeness of PPI stakeholder input.

### Flexibility and adaptability

#### Optimising accessibility

Many studies reported the attrition of PPI stakeholders, particularly care home staff due to their demanding work schedules, sickness and differing priorities [49–51]. This high staff turnover resulted in irregularity in their involvement [30, 35, 44]. To attain an effective PPI approach in care home research, adaptations to the research schedule were required to sufficiently incorporate care home staff within research [41, 45].

#### Tailoring communication methods

To sustain collective involvement of PPI stakeholders, strategic and flexible ways of communication need to be adopted. Intentional dialogue pathways such as email, telephone or letters were instrumental methods of interaction [26, 32, 48, 51]. Creative methods such as role play and parallel workshops were utilised [37, 42, 46] and virtual meetings once a rapport was established [25, 31]. This approach differs to conventional dialogue pathways as it provides a platform for PPI stakeholders to express their interpretations of research findings and contribute to the design process in an informal and relaxed setting; thus, promoting collaboration and a transparent passage of information with the research team. Some studies recognised the challenges of digital literacy in older adults and designated an ‘embedded researcher’ who had an ‘open-door’ policy to facilitate the concerns of the PPI stakeholders [26, 32, 40, 45].

### Resources and wider support

#### Additional funding

A financial budget that incorporates the costly elements of PPI enhanced the effectiveness of PPI in one study [26]. Some studies covered expenses for travel, printing, telephone use [34, 50] and the cost of time [32, 33, 49]. Other studies offered an honorarium payment for participation [38, 45] in one study, staff involvement was part of a secondment arrangement funded by government [29]; whilst other studies used national guidelines for user involvement to stipulate the value of travel expenses and honorariums that PPI stakeholders should receive [33]. But not all PPI stakeholders were rewarded [34] and the payment amount was not usually divulged within the reports [43, 45].

#### Recognising training needs

An effective PPI approach considered the use of training programmes to help PPI stakeholders ‘find direction’ within the research [50]. Most studies focused on practical research skills including, data collection, analysis and interviewing [39, 41] which varied in length from 1 day programmes to several weeks [32, 34] and was conducted by specialists in the field [25, 40]. Opposingly, one study preferred the pre-existing skills, expertise and perspectives of the PPI stakeholders rather than academic training [26].

## Discussion

The findings from this review support a growing a body of literature that highlights the value PPI brings to research, improving its relevance and applicability [53–56]. Care home residents and staff have unique insights as co-researchers, their experiential knowledge providing valuable learning opportunities for academic researchers [4]. The authentic experiences of PPI stakeholders can encourage practice improvement and culture change within research where residents and staff become ‘professionalised users’ and ‘experts by experience’ within research [1, 2, 4, 14]. For this shift to occur with meaningful involvement between PPI stakeholders and academic staff, mutual partnerships and relationships need to be fostered which are flexible in power-sharing and decision-making [57]. Previous studies have identified effective approaches to the inclusion of diverse groups [58] and people receiving palliative and end of life care [59]. This review identified several factors specifically associated with effective PPI in care home research in addition to similar themes around gatekeeping, communication, and a lack of reporting of PPI activities.

Due to the lack of consistency of PPI reporting in included studies, and an absence of established methodological rigour, the role of PPI was variable according to the care establishment and research context. The transparency of the PPI process was variable particularly within nursing homes where PPI stakeholders were either selected according to a convenience sample and overall representation with limited explanation of the PPI recruitment process [29, 42, 47, 49]. This is juxtaposed to formal recruitment methods where stakeholders were encouraged to enrol via meetings, conferences and online platforms and were informed of the time commitments, duration of participation and their role in the project [30, 38, 48].

The degree of stakeholder collaboration varied significantly, often care home residents had limited involvement compared to other stakeholder groups. Key stakeholders referred to residents (older people and adults with disabilities), relatives, caregivers and representative organisations. However, due to power dynamics and polyvocal perspectives there were conflicts of interest resulting in the views of residents being overpowered by caregivers and representative organisations [27, 45]. Moreover, numerous studies have highlighted the challenges of ensuring broad representation of vulnerable adults in research due to physical and cognitive impairments which affect the level of participation [2, 4, 54]. These individual-led barriers to involvement are often reasons why care home residents are often excluded from studies [60] or only informally involved [3], whilst fitter and more independent residents tend to be more active PPI stakeholders [61].

The negative perceptions associated with care homes often limits residents’ engagement in research as sole participants; becoming the ‘researched’ as opposed to the ‘co-researchers’. This was highlighted in the study with younger adults [25] where despite being younger than 65 years old, their physical impairments of cerebral palsy, multiple sclerosis and muscular dystrophy limited the extent of their involvement in the research setup. Notably, only one study explored the role of PPI with younger adults in care homes which underscores the preconceived ideas of care home residents, their abilities and degree of involvement in research. Arguably, the disparities of PPI and research engagement within care homes extends beyond age but is instead bound by the societal perceptions of the catch-all term ‘care home’—of which more transparent research is needed to address.

The growing importance of equality, diversity and inclusivity in research offers a diverse perspective to recruitment strategies. Representation from those with varying educational attainment, gender, geography, and ethnicity provides a broader perspective on the issues affecting vulnerable adults [9, 10, 14]. Black et al. [14] had a diverse sample of PPI stakeholders with an equal split of male and females, three different ethnicity categories and a wide range of ages which created a welcoming research environment and improved research partnerships. Bindels et al. [4] noted gender and educational attainment influence the experience of ageing with differing health behaviours and outcomes. Consequently, an effective PPI approach will adopt appropriate recruitment processes to retrieve diverse personal perspectives. Additionally, conducting research in care homes with younger residents [25] may require alternative approaches to PPI as the challenges and approaches identified in this review may be specific to the physical and cognitive disabilities more often encountered by older people or may not be applicable to other care settings such as supported accommodation.

Care home residents can aid dissemination, research findings should be distributed to non-academic settings by PPI stakeholders who are sensitive to the tone of dissemination [1]. For example, Gridley et al. [62] involved people with dementia to produce a short film and a plain English summary to disseminate the results. The PPI stakeholders included people with dementia, care staff and family caregivers. Similarly, Bethell et al. [63] purposefully engaged people with dementia in developing surveys, priority-setting and implementing recommendations. This continuum of involvement encourages democratisation of research that is inclusive and appropriately aligned to the needs of PPI stakeholders [2, 4].

The first-hand experiences and reflections of stakeholders’ regarding their role of PPI in care home research was not always explicitly stated. Some articles highlighted the perception of being ill-informed or unknowledgeable regarding the research concepts [50] whilst other articles evaluated the success of the PPI project through dialogue excerpts, detailing the dissemination attempts and outlining motivations for involvement [43, 44, 46, 48]. Whereas the perceived impact and the evaluation of PPI outcomes was implied for most articles [25, 36, 39, 41, 42, 45, 47]. Incorporating critical and collective reflections of PPI stakeholders and researchers within research projects will develop a transparent working environment that promotes collaboration between science and practice [35].

### Strengths and limitations

This review was prospectively reported on PROSPERO. The inclusion criteria provided a platform for care home residents who are often underrepresented in research, whilst recognising that the perspectives and experiences of a wider range of stakeholders are often valuable to include. However, challenges were encountered during the screening process as PPI is not always being reported in the title, abstract or study aims which may have led to relevant studies not being included. Additionally, the lack of clear definitions around involvement, participation, engagement, and co-production made it difficult to differentiate between the role of PPI stakeholders within the research design. Consequently, more research is required to characterise key terminology along the PPI continuum and to develop tools to appraise the quality of articles reporting PPI activities or approaches. The variable quality of PPI reporting has been widely reported elsewhere and led to the development of reporting frameworks such as GRIPP2 [64].

Our findings are supported by a scoping review which was published following our review and which focused on mapping co-production approaches to care home research for older adults [16]. As in our review which considered the wider spectrum of public involvement, the review of co-production approaches identified a broad range of stakeholder involvement and highlighted the importance of reciprocal relationships and ensuring inclusive opportunities [16].

Due to resource limitations public involvement was not included in the research design, analysis or authorship of this study which, if incorporated, would have enhanced the outcomes and conduct of the study and provide opportunities to apply the research findings into practice. We were similarly limited to English language studies which impacted the representativeness of the sample. Notably, most of the studies were conducted prior to the COVID-19 pandemic during which there were seismic shifts in the nature and mode of PPI activities. Other limitations included the risk of bias, with a single reviewer of full-text eligibility and data extraction and a sample independently reviewed by a second reviewer. This may have resulted in subjective assessment and key methodological concerns remaining unidentified.

### Practice Implications

These findings unify current understanding of incorporating care home residents as PPI members within research. Utilising evidence-based insights with appropriate adjustments to PPI methods (Fig. 2), care home residents and marginalised groups can engage within research, resulting in findings that are more relevant to the target population.

We have used the findings from this review to develop practical recommendations that will support more inclusive and effective PPI in care home research (Table 4).

**Table 4:** Practical recommendations to initiate effective PPI approaches within care home research

Overarching themes	Subthemes	Practical recommendations
*Valuing stakeholders’ perspectives* There must be a clear recognition that the lived experiences and personal viewpoints of PPI stakeholders are pivotal to the research design and outcomes, including the dissemination and impact to clinical practice	Amplified voice Experts by experience Achieving meaningful impact	Residents should be viewed as ‘experts by experience’ and supported to make practical suggestions to implement changes that are appropriately aligned to their needs (e.g. co-designing affirmative titles such as ‘Sages’ or ‘VOICES’ to amplify their involvement in the project) Researchers should seek opportunities for residents and other stakeholders to aid dissemination of the findings within non-academic settings (e.g. presenting to care home managers within multi-disciplinary meetings)
*Inclusivity and transparency* Creating a research environment of inclusivity and transparency enables diverse stakeholder groups to be involved in research; via a spectrum of involvement which provides a safe communicative space for PPI stakeholders to build a rapport with the research team	Spectrum of involvement Safe communicative space	Researchers should undertake stakeholder mapping to identify who should be included and provide opportunities across the spectrum of involvement (e.g. co-produce and monitor involvement strategies to ensure a diverse proportion of stakeholders are involved) Research teams should ensure inclusive environments, cultures and practices which recognises that stakeholders will have individual communication needs (e.g. providing materials in large print and audio formats) Researchers should recognise that care home residents are not a heterogenous group but have diverse backgrounds and opinions which provide invaluable perspectives which positively impacts research (e.g. identify the skills, expertise and experiences of the stakeholders from the beginning of the project)
*Multi-faceted research context* An effective PPI approach considers the complexity of the research topic in relation to the existing knowledge of PPI stakeholders, balances the power dynamic hierarchies and encourages a representative recruitment approach	Balancing power dynamics Navigating the research topic Appropriate recruitment process	Researchers should recognise and address potential power imbalances, such as hosting separate PPI meetings with layperson briefings, so stakeholders remain integrated with the research development Stakeholders should be provided with opportunities to engage in research following tailored recruitment strategies (e.g. advertisements within care homes, partnering with representative organisations)
*Flexibility and adaptability* An iterative and flexible approach to the methods of involvement, accommodates the diverse needs of PPI stakeholders and adapts to the preferred mode of communication and optimises accessibility	Optimising accessibility Tailoring communication methods	Stakeholders should be provided with alternative dialogue pathways (virtual meetings, email, telephone, or letters) to engage with the research team, and adapted as appropriate Researchers should ensure that the communication and support needs of stakeholders are assessed and addressed (e.g. develop tailored communication strategies using tools such as the Consent Support Tool and use of reflection throughout the project)
*Resources and wider support* The ability to tailor training to PPI stakeholders’ needs and offer additional financial aid, enhances the scale of collaboration, and impacts the research design	Additional funding Recognising training needs	Funders of health and care research should recognise that additional resources are needed to undertake effective PPI in care home research and ensure appropriate funding is available to support relationship-building Researchers should undertake training to develop appropriate research-specific skills (e.g. data collection and interviewing people with cognitive impairment)Research organisations should provide training sessions for research teams about how to confidently engage with people with physical impairments and additional needs (e.g. communication and role play workshops)

*PPI* patient and public involvement

### Future research

We have used the evidence-based PPI guidelines to propose practical recommendations which can be applied in future care home research. Further exploration is needed to develop strategies for involving residents with greater support needs and time-pressured care home staff within research. Strategies are also needed which extend beyond the traditional methods of PPI to incorporate more innovative approaches within different study designs, whilst establishing appraisal tools to adequately measure and report the quality of PPI.

## Conclusion

People living in care homes often require the highest and most complex level of care, whose needs are met by a large and diverse workforce. An effective PPI approach transforms the view of residents and staff as consumers with a passive voice to owners of research with active and valuable participation in the creation of knowledge. This is achieved by careful consideration of five person-centred factors: valuing stakeholders’ perspectives, ensuring inclusivity and transparency, taking account of the multi-faceted research context, maintaining flexibility and adaptability whilst utilising resources and wider support. It is hoped that the practical recommendations given can transform the research culture and create a research environment that is representative and accessible to this under-served group regardless of age, experience, or health status.

## Data Availability

Supplementary materials and data extracted can be obtained from the authors on reasonable request.

## Appendix 1. Search strategy

Search syntax used for bibliographic database searches*

**Table:**

Public involvement in research		Care homes	Attitudes and approaches
Patient* adj3 public Involve* public adj3 involve* Patient* adj3 Engage* Public adj3 engage* Patient* adj3 Participat* Public adj3 Participat* Participat* research Patient* advocacy Patient* adj3 Partner* Public adj3 partner*	Patient* adj3 consult* Public adj3 consult* Patient* adj3 Evaluat* Public adj3 evaluat* Consumer* adj3 Participat* Service user* adj3 research Service user*adj3 involve* Stakeholder* adj3 participat* Stakeholder* adj3 involve	Assisted living Care home* Long-term care Facil* Long term care facil* Nursing home* Residential home*	Approach* Attitude* Framework* Method* Model* Tool* Collaborat* Consult* Co-produc* Coproduc*

*The search syntax was developed for MEDLINE and adapted for SCOPUS and CINAHL databases

## Appendix 2. Data extraction form

**Table:**

*Study details*
Date of review (reviewer)
RefID
First author
Year of publication
Article title
Journal name
Region (setting/country)
Type of care establishment
Funding body
*Study topic*
Study topic
Objectives and aims
*Methods*
Number of homes involved
Study duration
Study design
*PPI types and aims*
PPI terminology
Type of PPI
Aims of PPI
Decision or advice PPI are involved with
*PPI and interactions*
Groups of people involved
Inclusion/exclusion criteria
Amount of PPI people
Demographics of PPI (age, gender, ethnicity, relationship)
Interaction between PPI groups
Retention of PPI people
*Recruitment and motivation*
Recruitment of PPI members
Motivation of PPI people to become involved
*Stages of PPI*
Stages of project with PPI
Frequency of Involvement

## Abbreviations

CHIG: Care Home Interest Group
LGBT: Lesbian, gay, bisexual, and transgender
MMAT: Mixed methods appraisal tool
NIHR: National Institute for Health Research
PIRg: Public Involvement in Research Group
POPPs: Partnerships for older people projects
PPI: Public involvement
PRISMA: Preferred reporting items for systematic reviews and meta-analyses
SHARP: Seniors helping as research partners
SPIDER: Setting, phenomenon of interest, design, evaluation, research design
VOICES: Voices of individuals, family and friend caregivers educating us
